# Real-world outcomes of immune checkpoint inhibitors as second-line therapy for extensive-stage small-cell lung cancer: a multicenter retrospective analysis

**DOI:** 10.3389/fimmu.2025.1658017

**Published:** 2025-09-19

**Authors:** Jiao Zhang, Jia-xing Guo, Ping Li, Xiao-ye Jin, Yan Wang, Lu-jun Zhao

**Affiliations:** ^1^ The Third Department of Medical Oncology, General Hospital of Ningxia Medical University, Yinchuan, Ningxia, China; ^2^ Department of Radiation Oncology, Tianjin Medical University Cancer Institute and Hospital, National Clinical Research Center for Cancer, Tianjin Key Laboratory of Cancer Prevention and Therapy, Tianjin’s Clinical Research Center for Cancer, Tianjin, China; ^3^ Department of Radiation Oncology, The Affiliated Hospital of Inner Mongolia Medical University, Hohhot, Inner Mongolia Autonomous Region, China; ^4^ Department of Pharmacy, General Hospital of Ningxia Medical University, Yinchuan, Ningxia, China; ^5^ College of Clinical Medical, Ningxia Medical University, Yinchuan, Ningxia, China

**Keywords:** extensive-stage small cell lung cancer, immune checkpoint inhibitors, second-line therapy, survival, prognosis

## Abstract

**Background:**

There is limited evidence concerning real-world efficacy of second-line (2L) treatment with immune checkpoint inhibitors (ICIs) in extensive-stage small-cell lung cancer (ES-SCLC). In this study, we evaluated the efficacy of 2L-ICIs therapy in patients with ES-SCLC.

**Methods:**

In this retrospective study, we included patients with ES-SCLC who experienced disease progression following first-line (1L) therapy and received 2L treatment between March 2019 and December 2023. The primary endpoint of this study was progression-free survival (PFS), and the secondary endpoints included safety, the objective response rate (ORR), the disease control rate (DCR), and overall survival (OS). Survival analyses were conducted using Kaplan-Meier curves. One-to-one propensity score matching (PSM) was used to reduce confounding. Univariate and multivariate Cox regression analyses were conducted to identify factors associated with PFS and OS.

**Results:**

We included 496 patients in this study; among them, 200 patients were in the 2L-ICIs group and 296 patients were in the 2L-non-ICIs group. The 2L-ICIs group demonstrated significantly longer PFS than the 2L-non-ICIs group (median PFS: 4.13 vs. 2.70 months; p < 0.001), and this benefit persisted after PSM (median PFS: 4.14 vs. 2.84 months; p < 0.001). The 2L-ICIs group also had a significantly higher ORR (ORR: 29.5% *vs*. 10.1%; p < 0.001) and DCR (DCR: 67.0% *vs*. 51.7%; p < 0.001). Treatment-related adverse events were comparable between the groups, with only one grade 3 rash reported in the 2L-ICIs group. Multivariate Cox regression identified liver metastases, the number of metastatic lesions, and the 1L-PFS as independent predictive factors for PFS.

**Conclusion:**

In this study, 2L-ICIs demonstrate significant clinical benefits with acceptable toxicity in ES-SCLC patients who progressed after 1L therapy, supporting their use as a clinically actionable option.

## 1 Introduction

Lung cancer is the leading cause of cancer incidence and cancer-related mortality worldwide, accounting for an estimated 2.5 million new cases (12.4%) and 1.8 million deaths (18.7%) annually (1). However, significant variation in lung cancer incidence and mortality rates exists across different regions of the world, reflecting different patterns of tobacco smoking, exposure to environmental risk factors, and genetics (2). In this context, lung cancer poses a particularly severe public health challenge in China, accounting for a disproportionately high share of the global burden. Recent data from the Global Burden of Disease (GBD) study provided strong evidence for this occurrence. China accounts for 41.0% of new lung cancer cases and 40.4% of lung cancer-related deaths worldwide (3); these percentages are substantially greater than those reported in all other nations. Epidemiological trends in China diverge markedly from those in high-income countries. For example, the United States has experienced a decline in both incidence and mortality since the 1990s through multifaceted interventions, including comprehensive tobacco control, early screening implementation, and therapeutic advances (4). Small cell lung cancer (SCLC) is the most aggressive lung cancer subtype; it is strongly associated with tobacco exposure and accounts for about 15% of all new diagnoses (5, 6). About 70% of SCLC patients present with extensive-stage disease at initial diagnosis (7). The prognosis of extensive-stage SCLC (ES-SCLC) is extremely poor, with a median overall survival (OS) of only 6–10 months (8) and a five-year survival rate of less than 2% (9).

Progress concerning the treatments for SCLC over three decades has been limited (10). Platinum-based doublet chemotherapy is the standard first-line (1L) therapy for ES-SCLC (11). The recent integration of immune checkpoint inhibitors (ICIs) with platinum-based chemotherapy has become the new 1L standard, extending the median OS by about two months (12, 13). Subsequent phase III trials evaluating novel ICIs (e.g., serplulimab, adebrelimab, and tislelizumab) have demonstrated greater efficacy, achieving median OS ranging from 15.3 to 15.5 months (14–16). Despite high initial sensitivity to 1L chemotherapy, acquired resistance mediated by tumor evolution under selective therapeutic pressure leads to near-universal progression of disease (17). Consequently, the therapeutic landscape for second-line (2L) and subsequent treatments remains severely constrained. The 2L therapeutic agents currently used, including standard agents such as topotecan, the newer DNA-alkylating agent lurbinectedin (18), and the DLL3-targeted bispecific T-cell engager tarlatamab (19), are not easily accessible and have safety concerns (20).

The efficacy of ICIs in the 2L treatment of ES-SCLC remains controversial. Previous phase II/III trials evaluating ICI monotherapy or dual immunotherapy combinations have failed to demonstrate significant survival benefits (21–24). However, several studies have suggested that ICIs may provide clinical benefits in 2L therapy (25–28). Consequently, strong evidence is needed to support the efficacy of 2L ICIs after disease progression following 1L therapy in ES-SCLC patients in clinical decision-making. The safety issues associated with 2L ICIs also need to be investigated. In this multicenter study, we evaluated the efficacy and safety of 2L ICIs for treating ES-SCLC.

## 2 Materials and methods

### 2.1 Patients

Patients with ES-SCLC who received 2L therapy at Tianjin Medical University Cancer Institute and Hospital, General Hospital of Ningxia Medical University, and The Affiliated Hospital of Inner Mongolia Medical University between March 2019 and December 2023 were retrospectively included in this study. The inclusion criteria were as follows: (1) All patients must be ≥ 18 years old. (2) SCLC was confirmed via pathological or cytological examinations. (3) Patients presented with extensive-stage disease at initial diagnosis, defined by the NCCN criteria as extension beyond the ipsilateral hemithorax, including malignant pleural or pericardial effusion or hematogenous metastases. (4) Patients showed disease progression following 1L therapy. (5) Patients must have a baseline corticosteroid dose ≤10 mg/day of prednisone equivalent and adequate marrow and organ function. (6) Patients for whom complete clinical medical records were available. Exclusion criteria were as follows: (1) Histopathology showing combined SCLC with other cellular components (e.g., adenocarcinoma or large cell carcinoma). (2) Patients with a history of other concurrent malignancies. (3) Patients with active autoimmune disease, immune deficiency, hepatitis B virus (HBV) or hepatitis C virus (HCV) infection, or active tuberculosis (TB). (4) Patients with symptomatic brain metastases.

Patients were stratified into 2L-ICIs and 2L-non-ICIs groups based on whether ICIs were added to 2L therapy.

### 2.2 Data collection

Baseline clinical characteristics at diagnosis, including gender, age, smoking status (never/former or current smoker), Eastern Cooperative Oncology Group (ECOG) performance status (PS), and metastatic sites, were extracted from electronic health records.

### 2.3 Outcomes and assessments

The primary outcome was progression-free survival (PFS), defined as the time from the initiation of 2L therapy to disease progression or death due to any cause. The secondary endpoints were safety, the objective response rate (ORR), the disease control rate (DCR), and OS. Tumor response was evaluated by conducting contrast-enhanced computed tomography of the chest and upper abdomen, as well as contrast-enhanced magnetic resonance imaging of the brain, initially at baseline and then after every second treatment cycle (6–8 weeks). Tumor response was assessed according to the Response Evaluation Criteria in Solid Tumors (RECIST v1.1). The best overall response categories included complete response (CR), partial response (PR), stable disease (SD), and progressive disease (PD). The ORR was defined as the proportion of patients who achieved CR or PR. DCR was defined as the proportion of patients who achieved CR, PR, or SD. OS was defined as the time from the initiation of 2L therapy to the date of death due to any cause or the last day of follow-up. Treatment-related adverse events (TRAEs) were graded using the Common Terminology Criteria for Adverse Events version 5.0 (CTCAE v5.0). The last date of follow-up for the study was January 10, 2025. The follow-up time was defined from the initiation of 2L therapy until death or the last follow-up date, whichever occurred first.

### 2.4 Statistical analysis

Baseline characteristics for categorical variables are presented as frequencies and percentages. The Chi-square test or Fisher’s exact test was conducted to compare categorical variables between the two groups. To minimize potential confounding factors, one-to-one propensity score matching (PSM) was performed with a caliper width of 0.02. The Kaplan-Meier method was used to estimate PFS and OS, and differences between groups were assessed by conducting the log-rank test. Univariate and multivariate Cox proportional hazards regression models were used to identify factors associated with survival outcomes. Variables with P < 0.15 in the univariate analysis were included in the multivariate model. The hazard ratio (HR) was reported along with the 95% confidence interval (CI). While conducting subgroup analyses, we used an unstratified Cox proportional hazards model with 2L treatment as a covariate. The results were considered to be statistically significant at P < 0.05 (two-sided). All statistical analyses were conducted using SPSS version 25.0 (IBM Corp., Armonk, NY, USA).

## 3 Results

### 3.1 Baseline characteristics

A total of 496 ES-SCLC patients who received 2L therapy were included in this study. The cohort had a median age of 62 years (range: 19–87), with predominant clinical characteristics, including male gender (79.6%), a smoking history (71.0%), and ECOG PS <2 (79.2%). Among the patients, lung (58.9%), bone (31.3%), brain (20.8%), and liver (20.2%) metastases were observed, with 72.0% of patients having ≥3 metastatic sites. ICIs were administered as a 1L treatment in 42.5% of the patients. After PSM, the baseline characteristics were balanced between the groups (**Table 1**).

**Table 1:** Baseline demographic and clinical characteristics of the patients.

Characteristics	Before PSM		P	After PSM		P
	2L-ICIs (n = 200)	2L-non-ICIs (n = 296)		2L-ICIs (n = 162)	2L-non-ICIs (n = 162)	
	No. (%)	No. (%)		No. (%)	No. (%)	
Gender			0.397			0.497
Male	163(81.5)	232(78.4)		130(80.2)	125(77.2)	
Female	37(18.5)	64(21.6)		32(19.8)	37(22.8)	
Age			0.239			0.250
≥65	80(40.0)	103(34.8)		65(40.1)	55(34.0)	
<65	120(60.0)	193(65.2)		97(59.9)	107(66.0)	
Smoking status			0.412			0.707
Yes	146(73.0)	206(69.6)		117(72.2)	120(74.1)	
No	54(27.0)	90(30.4)		45(27.8)	42(25.9)	
ECOG PS			0.916			0.109
≥2	42(21.0)	61(20.6)		30(18.5)	42(25.9)	
<2	158(79.0)	235(79.4)		132(81.5)	120(74.1)	
Lung metastases			0.125			1.000
Yes	126(63.0)	166(56.1)		96(59.3)	96(59.3)	
No	74(37.0)	130(43.9)		66(40.7)	66(40.7)	
Bone metastases			0.139			0.544
Yes	70(35.0)	85(28.7)		46(28.4)	51(31.5)	
No	130(65.0)	211(71.3)		116(71.6)	111(68.5)	
Brain metastases			0.217			0.670
Yes	47(23.5)	56(18.9)		32(19.8)	29(17.9)	
No	153(76.5)	240(81.1)		130(80.2)	133(82.1)	
Liver metastases			0.286			0.886
Yes	45(22.5)	55(18.6)		30(18.5)	29(17.9)	
No	155(77.5)	241(81.4)		132(81.5)	133(82.1)	
Number of metastatic lesions			0.101			0.617
≥3	152(76.0)	205(69.3)		116(71.6)	120(74.1)	
<3	48(24.0)	91(30.7)		46(28.4)	42(25.9)	
1L-ICIs			<0.001			0.912
Yes	119(59.5)	92(31.1)		81(50.0)	82(50.6)	
No	81(40.5)	204(68.9)		81(50.0)	80(49.4)	
1L-PFS (months)			0.177			0.822
≥6	125(62.5)	167(56.4)		95(58.6)	93(57.4)	
<6	75(37.5)	129(43.6)		67(41.4)	69(42.6)	

### 3.2 Treatment

In the 2L-ICIs group (n = 200), all patients received ICI-based regimens, which included ICI-chemotherapy combinations (64.5%, n = 129), ICI-antiangiogenic therapy (22.5%, n = 45), triple therapy (ICI + chemotherapy + anti-angiogenic agent; 10.5%, n = 21), and ICI monotherapy (2.5%, n = 5). The ICI agents used were atezolizumab (52.5%, n = 105), durvalumab (27.0%, n = 54), serplulimab (12.5%, n = 25), tislelizumab (4.5%, n = 9), and adebrelimab (3.5%, n = 7). The median number of 2L ICIs therapy cycles was 3 (range: 1–16). In the 2L-non-ICIs group (n = 296), treatment regimens consisted of chemotherapy (82.1%, n = 243), including platinum-based combinations (e.g., etoposide/platinum, irinotecan/platinum), irinotecan monotherapy, taxanes, temozolomide, and the CAV regimen (cyclophosphamide, adriamycin, and vincristine), as well as anti-angiogenic therapy (17.9%, n = 53; anlotinib monotherapy or combination with chemotherapy). These patients received a median of two cycles of therapy (range 1–28).

### 3.3 Efficacy

**Figure 1:**
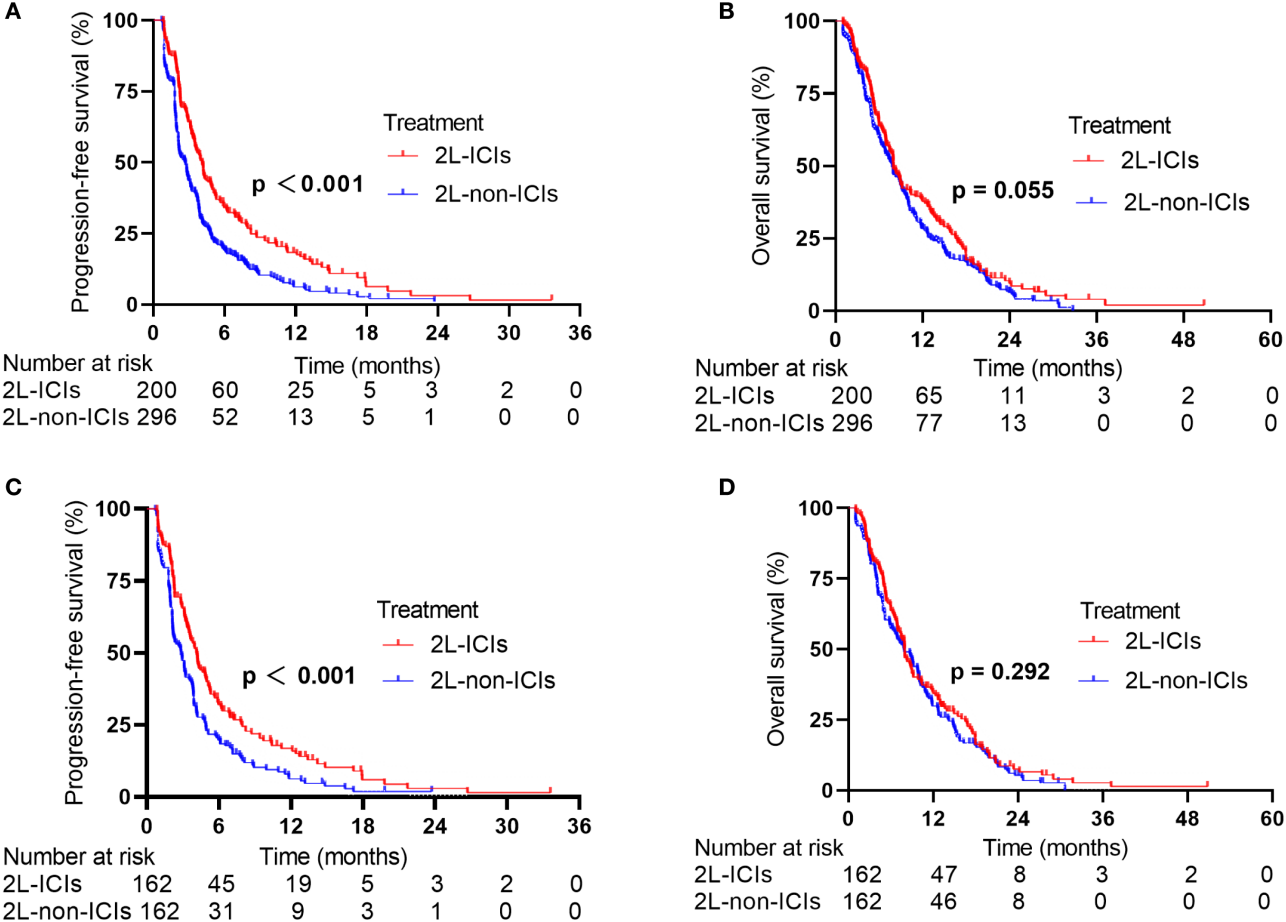
PFS and OS between the 2L-ICIs group and the 2L-non-ICIs group before and after PSM. **(A)** PFS before PSM, **(B)** OS before PSM, **(C)** PFS after PSM **(D)** OS after PSM.

In the whole population, the median follow-up time was 19.7 months, with median PFS and OS values of 3.12 and 7.90 months, respectively. In the 2L-ICIs group, the median PFS was 4.13 months (95% CI: 3.63–4.63), whereas it was 2.70 months (95% CI: 2.29–3.11) in the 2L-non-ICIs group (HR 0.62, 95% CI 0.51–0.75; p < 0.001; **Figure 1A**). The six-month PFS rates were 34.1% and 19.1%, and the one-year PFS rates were 18.3% and 6.2%, respectively. In terms of OS, although no significant difference was observed between the 2L-ICIs and 2L-non-ICIs groups, a slight improvement in OS was noted in the 2L-ICIs group. The median OS was 7.98 months (95% CI: 7.02–8.94) in the 2L-ICIs group versus 7.80 months (95% CI: 6.79–8.81) in the 2L-non-ICIs group (HR 0.83, 95% CI 0.68–1.00; p = 0.055; **Figure 1B**), with one-year OS rates of 37.8% and 28.4% and two-year OS rates of 9.5% and 6.3%, respectively.

After 1:1 PSM, the overall median PFS was 3.45 months, and the median OS was 7.95 months. The median PFS was 4.14 months (95% CI: 3.62–4.67) in the 2L-ICIs group versus 2.84 months (95% CI: 2.19–3.49) in the 2L-non-ICIs group (HR 0.65, 95% CI 0.51–0.82; p < 0.001; **Figure 1C**). The six-month PFS rates were 32.1% and 19.8%, and the one-year PFS rates were 16.9% and 6.3%, respectively. The median OS was 7.96 months (95% CI: 7.01–8.92) in the 2L-ICIs group and 7.87 months (95% CI: 6.13–9.61) in the 2L-non-ICIs group (HR: 0.88, 95% CI: 0.70–1.11; p = 0.292; **Figure 1D**). The one-year OS rates were 34.4% and 29.9%, and the two-year OS rates were 7.6% and 5.4%, respectively.

### 3.4 Response

The ORR and DCR for the entire cohort were 17.9% and 57.9%, respectively. In the 2L-ICIs group, the ORR was 29.5%, and the DCR was 67.0%, whereas in the 2L-non-ICIs group, the ORR and DCR were 10.1% and 51.7%, respectively. These differences were significant for both the ORR (p < 0.001) and DCR (p < 0.001). After PSM, the ORR was 29.6% in the 2L-ICIs group versus 8.0% in the 2L-non-ICIs group, and the DCR was 67.3% versus 51.9%, respectively, with continued significance for the ORR (p < 0.001) and DCR (p = 0.005) (**Table 2**).

**Table 2:** Responses to second-line therapy.

Response	Before PSM		P	After PSM		P
	2L-ICIs (n = 200)	2L-non-ICIs (n = 296)		2L-ICIs (n = 162)	2L-non-ICIs (n = 162)	
	No. (%)	No. (%)		No. (%)	No. (%)	
CR	0	0		0	0	
PR	59(29.5)	30(10.1)		48(29.6)	13(8.0)	
SD	75(37.5)	123(41.6)		61(37.7)	71(43.8)	
PD	66(33.0)	143(48.3)		53(32.7)	78(48.2)	
ORR	59(29.5)	30(10.1)	<0.001	48(29.6)	13(8.0)	<0.001
DCR	134(67.0)	153(51.7)	<0.001	109(67.3)	84(51.9)	0.005

### 3.5 Safety

The incidence of TRAEs was comparable between the two groups. In the 2L-ICIs group, the immune-related adverse events (irAEs) observed included hypothyroidism, rash, pneumonitis, diarrhea, and adrenal insufficiency. Only one case of grade 3 rash occurred in this group. Most adverse events were manageable and generally reversible with standard clinical interventions (**Table 3**).

**Table 3:** TRAEs between the two groups.

TRAEs	2L-ICIs (n = 200)		2L-non-ICIs (n = 296)		P
	N	%	N	%	
Hematologic toxicities	111	55.5	163	55.1	0.924
G3/4 hematologic toxicities	32	16.0	47	15.9	0.971
Gastrointestinal toxicities	93	46.5	132	44.6	0.676
G3/4 gastrointestinal toxicities	22	11.0	34	11.5	0.867
Hepatic toxicities	76	38.0	97	32,8	0.231
G3/4 elevated ALT/AST	11	5.5	19	6.4	0.674
Hypothyroidism	14	7.0	0	0	<0.001
Rash	17	8.5	0	0	<0.001
G3 rash	1	0.5	0	0	0.403
Pneumonitis	4	2.0	0	0	0.026
Diarrhea	3	1.5	0	0	0.065
Adrenal insufficiency	3	1.5	0	0	0.065

### 3.6 Cox regression analysis for PFS and OS

For the 2L-ICIs population, we further investigated the risk factors affecting PFS. The results of the multivariate Cox regression analysis revealed that baseline liver metastases and the number of metastatic lesions were independent factors associated with worse PFS, and that 1L-PFS was an independent factor associated with favorable PFS (**Table 4**).

**Table 4:** Univariate and multivariate Cox regression analyses for PFS.

Characteristics	Univariate analysis		Multivariate analysis	
	HR, 95%CI	P value	HR, 95%CI	P value
Gender	1.061(0.726–1.552)	0.759		
Male				
Female				
Age	1.041(0.760–1.426)	0.802		
≥65				
<65				
Smoking status	1.126(0.802–1.579)	0.493		
Yes				
No				
ECOG PS	1.186(0.818–1.718)	0.368		
≥2				
<2				
Lung metastases	1.326(0.961–1.829)	0.086	1.245(0.829–1.868)	0.290
Yes				
No				
Bone metastases	1.139(0.828–1.568)	0.424		
Yes				
No				
Brain metastases	1.005(0.696–1.451)	0.978		
Yes				
No				
Liver metastases	2.109(1.469–3.027)	<0.001	1.782(1.201–2.643)	0.004
Yes				
No				
Number of metastatic lesions	1.886(1.295–2.748)	<0.001	1.831(1.151–2.912)	0.011
≥3				
<3				
1L-ICIs	0.971(0.712–1.325)	0.854		
Yes				
No				
1L-PFS (months)	0.652(0.478–0.890)	0.007	0.723(0.527–0.990)	0.043
≥6				
<6				

For OS, baseline liver metastases was identified as an independent factor associated with worse OS. In contrast, 1L-PFS was identified as an independent factor associated with favorable OS in the multivariate analysis (**Table 5**).

**Table 5:** Univariate and multivariate Cox regression analyses for OS.

Characteristics	Univariate analysis		Multivariate analysis	
	HR, 95%CI	P value	HR, 95%CI	P value
Gender	1.163(0.795–1.702)	0.437		
Male				
Female				
Age	1.145(0.837–1.566)	0.398		
≥65				
<65				
Smoking status	1.006(0.717–1.412)	0.971		
Yes				
No				
ECOG PS	1.216(0.839–1.764)	0.301		
≥2				
<2				
Lung metastases	1.336(0.968–1.844)	0.078	1.161(0.764–1.764)	0.485
Yes				
No				
Bone metastases	1.139(0.827–1.570)	0.425		
Yes				
No				
Brain metastases	1.049(0.726–1.517)	0.798		
Yes				
No				
Liver metastases	2.058(1.442–2.939)	<0.001	1.816(1.227–2.688)	0.003
Yes				
No				
Number of metastatic lesions	1.759(1.209–2.559)	0.003	1.591(0.996–2.543)	0.052
≥3				
<3				
1L-ICIs	0.840(0.617–1.144)	0.268		
Yes				
No				
1L-PFS (months)	0.618(0.453–0.843)	0.002	0.670(0.489–0.917)	0.013
≥6				
<6				

### 3.7 Subgroup analysis

**Figure 2:**
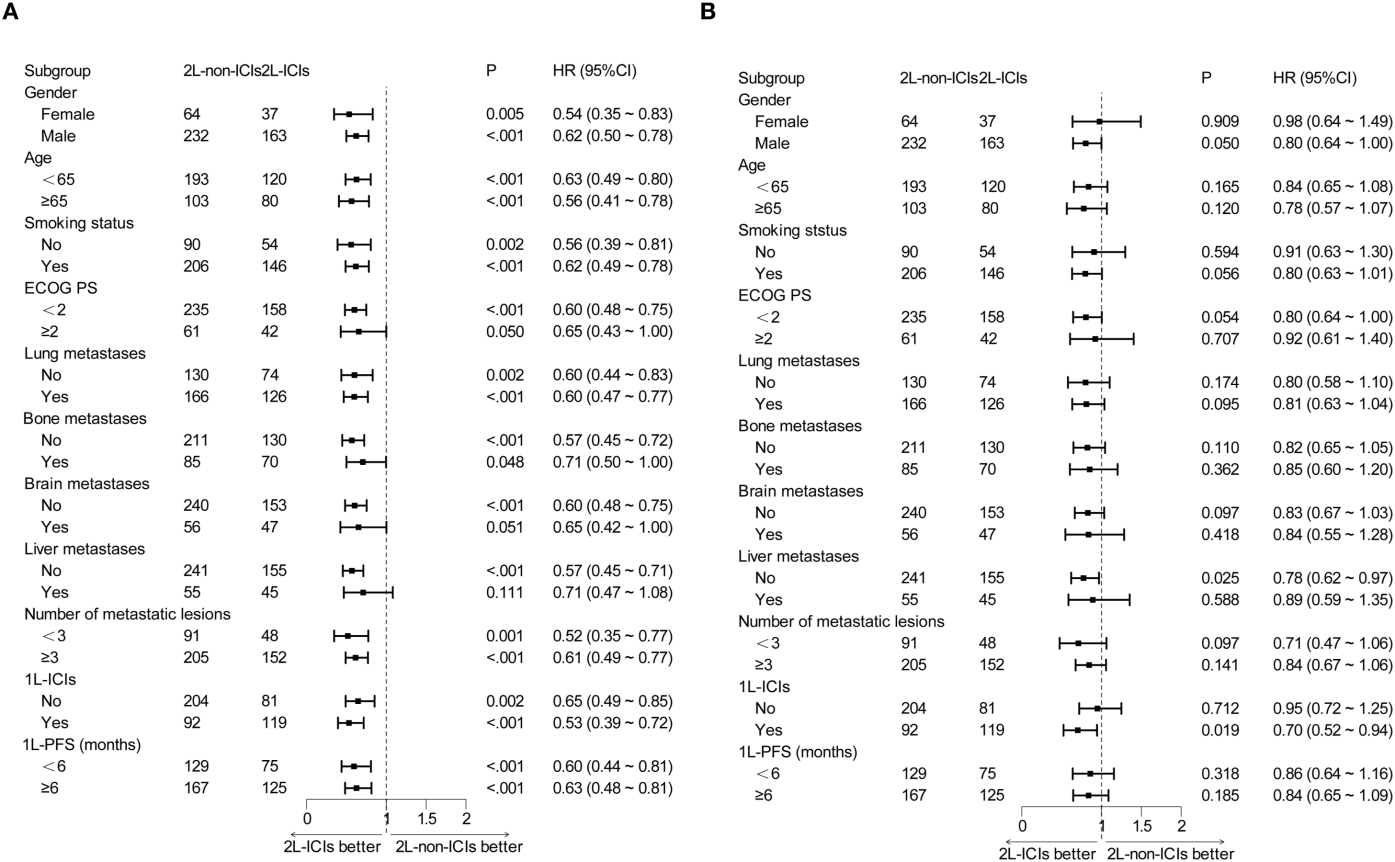
Subgroup analysis of prognostic factors for **(A)** PFS and **(B)** OS in the whole population.

Prespecified subgroup analyses stratified by baseline characteristics were performed. In most subgroups, 2L-ICIs improved PFS (**Figure 2A**). However, the improvement in OS with 2L-ICIs was observed only in the subgroup without liver metastases (HR 0.78, 95% CI 0.62–0.97; p = 0.025) and in those where the subgroups received 1L ICIs (HR 0.70, 95% CI 0.52–0.94; p = 0.019) (**Figure 2B**).

**Figure 3:**
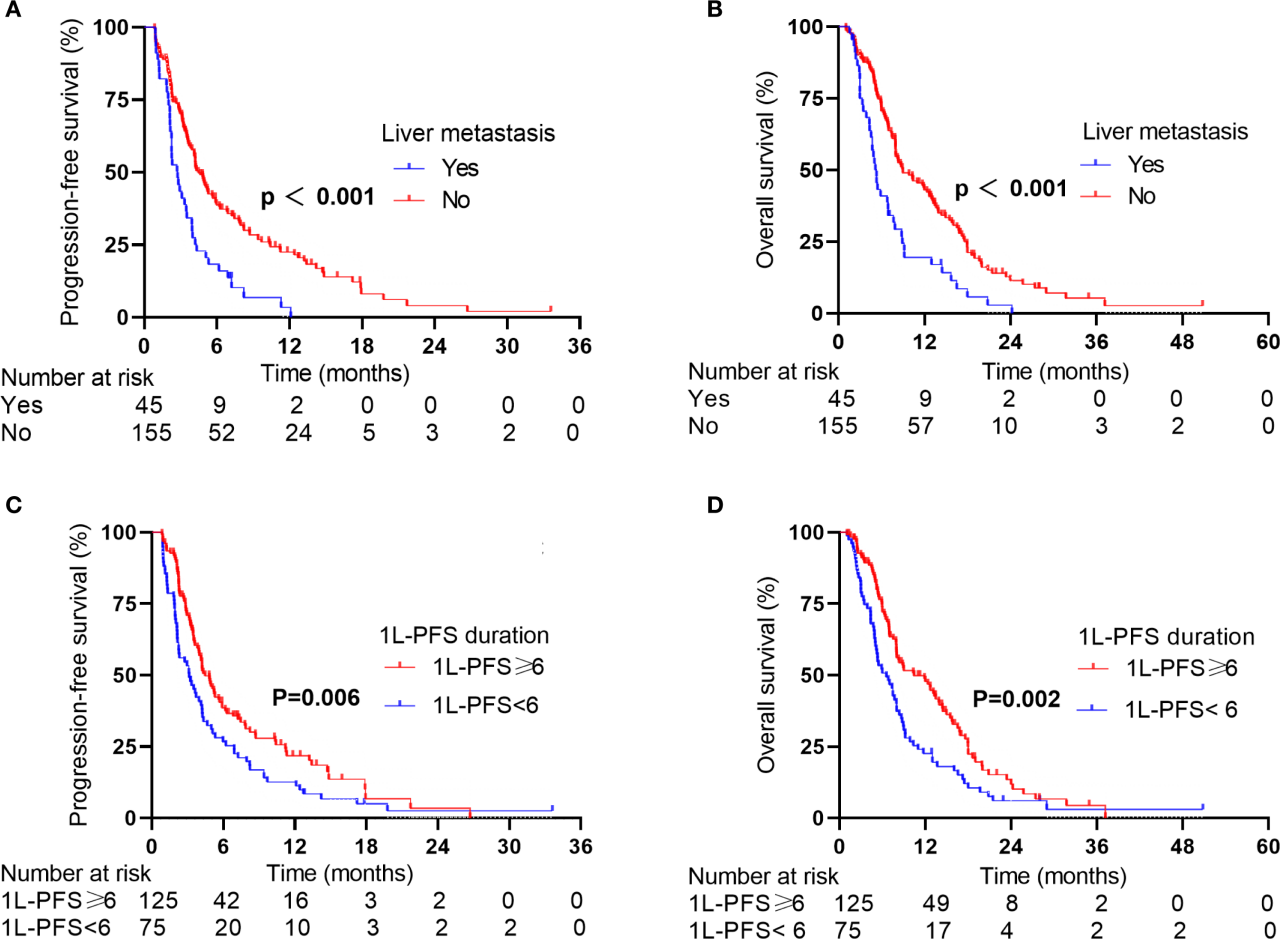
PFS and OS of patients in the 2L-ICIs group according to stratification factors. **(A)** PFS according to liver metastases status. **(B)** OS according to liver metastases status. **(C)** PFS according to the 1L-PFS duration. **(D)** OS according to the 1L-PFS duration.

Given the poor prognosis of patients with baseline liver metastases, we focused on patients with baseline liver metastases. In the 2L-ICIs cohort, patients with baseline liver metastases had shorter survival outcomes than those without baseline liver metastases. The median PFS was 2.74 months (95% CI: 2.03–3.45) versus 4.46 months (95% CI: 3.73–5.19) (p < 0.001, **Figure 3A**), with six-month PFS rates of 18.3% versus 38.8% and one-year PFS rates of 3.4% versus 22.5%. The difference was more pronounced in OS, where patients with liver metastases had a median OS of 5.32 months (95% CI: 4.54–6.10) compared to the median OS of 8.98 months (95% CI: 6.14–11.83) in patients without liver metastases (p < 0.001, **Figure 3B**). The one-year OS rates were 19.5% and 43.1%, and the two-year OS rates were 2.8% and 11.4%, respectively.

Considering 1L-PFS as an additional independent prognostic factor for survival, we further stratified patients by 1L-PFS to analyze survival differences. In the 2L-ICIs group, patients with a 1L-PFS of ≥6 months had a median PFS of 4.46 months (95% CI: 3.56–5.36) versus 3.13 months (95% CI: 1.90–4.36) in those with a 1L-PFS of <6 months (p = 0.006, **Figure 3C**), with corresponding 6-month PFS rates of 54.6% versus 27.2% and one-year PFS rates of 19.5% versus 17.2%. The survival benefit was evident in OS. Patients with a 1L-PFS of ≥6 months demonstrated a median OS of 10.43 months (95% CI: 6.57–14.29) compared to 6.54 months (95% CI: 4.73–8.35) in those with a 1L-PFS of <6 months (p = 0.002, **Figure 3D**). The one-year OS rates were 60.0% and 30.5%, and the two-year OS rates were 15.0% and 7.4%, respectively.

## 4 Discussion

Extensive-stage small-cell lung cancer is an aggressive malignancy with a poor prognosis, and treatment options after disease progression following 1L therapy remain limited. Currently available 2L treatments for ES-SCLC have minimal clinical benefit. The clinical evidence supporting ICIs efficacy in 2L therapy is insufficient. The cohort investigated in this study represents the largest real-world cohort evaluating the efficacy of 2L ICIs in ES-SCLC patients. We found that compared to 2L-non-ICIs therapy, 2L-ICIs therapy significantly improved PFS, ORR, and DCR while maintaining a favorable safety profile. The benefit of 2L-ICIs therapy persisted after PSM, a robust methodology minimizing confounding biases, confirming that our findings were reliable. This consistency in results highlights that ICIs can serve as a viable 2L treatment option for patients with ES-SCLC.

A study on the combination of camrelizumab and apatinib in ES-SCLC reported an ORR of 30%, a DCR of 70%, and a median PFS of 4.9 months (25). In our study, we found similar improvements in ORR and DCR with 2L ICIs, reinforcing the therapeutic benefit of ICIs for patients with ES-SCLC. Another phase II study examined the combination of sintilimab, anlotinib, and chemotherapy in patients with relapsed ES-SCLC and reported an ORR of 60%, a DCR of 76%, a median PFS of 6.0 months, and a median OS of 13.4 months (26). This study demonstrated considerably better clinical outcomes than our findings, probably because it exclusively enrolled patients who received the intensive triplet regimen of ICIs, anlotinib, and chemotherapy. The NCT02551432 trial, which analyzed the combination of pembrolizumab and paclitaxel, achieved a DCR of 80.7%, with a median PFS of 5.0 months and a median OS of 9.1 months (29). Our study similarly revealed that 2L ICIs significantly prolonged median PFS compared to 2L-non-ICIs, further supporting the efficacy of ICIs. Complementary real-world evidence confirmed these observations. A multicenter study reported that the combination of PD-1/PD-L1 inhibitors with anlotinib significantly outperformed paclitaxel monotherapy, with a higher DCR (80.5% *vs*. 54.5%; p = 0.005), longer median PFS (3.40 *vs*. 2.83 months; p = 0.02), and improved median OS (8.20 *vs*. 5.87 months; p = 0.048) (30). In another cohort of 103 patients, the administration of 2L ICIs significantly prolonged median PFS (4.4 months *vs*. 3.9 months, HR = 0.45, p = 0.001) and OS (10.0 months *vs*. 6.9 months, HR = 0.56, p = 0.015) compared to 2L-non-ICIs (31). Although a significant benefit of OS was absent in our cohort, clinically meaningful improvements in PFS, ORR, and DCR were observed, which is consistent with these findings. Our findings provide real-world evidence supporting the efficacy of treatment using 2L ICIs in clinically diverse ES-SCLC patients.

Potential explanations for the observed clinical benefits of 2L ICIs differ based on prior exposure to ICIs. For patients without prior exposure to IL ICIs, 2L ICIs block immune checkpoint pathways, abrogating suppressive signals in tumor-infiltrating T lymphocytes (TILs), thus identifying and destroying cancer cells (32). In contrast, for patients previously exposed to 1L ICIs, the sustained benefit from continued ICIs in 2L therapy is potentially attributable to the modification of the therapeutic regimen at this stage, such as alterations in the chemotherapy regimen or combinations of anti-angiogenic agents. These strategic therapeutic adaptations can contribute to the remodeling of the tumor immune microenvironment, thereby sustaining the efficacy of ICIs. However, the mechanisms underlying this observed benefit need to be further elucidated through dedicated mechanistic studies. Additionally, while our study demonstrated a significant improvement in PFS with 2L-ICIs compared to 2L-non-ICIs regimens, the absolute difference of 1.3 months is of limited clinical relevance. Consequently, the assessment of more effective and less toxic treatment strategies to optimize clinical outcomes remains a key focus of current research.

In this study, while 2L ICIs failed to significantly improve OS compared to 2L-non-ICIs, prespecified subgroup analyses confirmed a significant improvement in OS in patients without liver metastases in the 2L-ICIs group. This finding corroborates existing evidence indicating that cancer patients with liver metastases respond significantly more poorly to anti-PD-1 immunotherapy than those without liver metastases (24, 33–37). Consistent with these findings, our results demonstrated considerably poorer outcomes for patients with liver metastases. Both the median PFS (2.74 *vs*. 4.46 months) and OS (5.32 *vs*. 8.98 months) were about half the median PFS reported in patients without liver metastases (p < 0.001 for both). This survival disparity became more pronounced over time. The one-year PFS rate was only 3.4% in patients with liver metastases versus 22.5% in those without liver metastases, whereas a nearly fourfold difference was observed in the two-year OS rate (2.8% *vs*. 11.4%). Mechanistic insights from clinical and preclinical studies support these findings. Lee et al. proposed that liver tumors not only compromise intrahepatic immunity but also impair systemic antitumor immunity, potentially explaining the reduced efficacy of systemic anti-PD-1 treatment observed clinically (38). Supporting this mechanistic insight, Yu et al. reported that in preclinical models, liver metastases induce systemic loss of tumor-specific CD8+ T cells and abrogate the efficacy of immunotherapy, a phenomenon that mirrors systemic T-cell dysfunction and decreases the treatment response observed in patients with liver metastases (39). These results highlight the importance of performing routine assessment for liver metastases before initiating 2L ICIs therapy. For patients with liver metastases, 2L regimens combining ICIs with tumor microenvironment (TME)-modulating agents represent a strategic approach aimed at counteracting the underlying systemic immunosuppression. Such combinatorial strategies need to be validated by conducting prospective studies.

The results of subgroup analyses revealed significant improvements in OS with 2L ICIs in patients who received 1L ICIs. These findings are consistent with published results. Zhang et al. reported that continuing ICIs after progression provides a survival benefit for ES-SCLC patients without significantly increasing additional treatment-related toxicity. Their study revealed a longer median PFS (4.4 months *vs* 3.9 months, HR = 0.45, p = 0.001) and median OS (10.0 months *vs* 6.9 months, HR = 0.56, p = 0.015) in the 2L-ICIs group than in the 2L-non-ICIs group (31). Similarly, Shi et al. reported that atezolizumab continuation therapy had promising efficacy and manageable safety in ES-SCLC patients who progressed after 1L therapy, with a median PFS of 4.07 months (95% CI: 1.15–6.98) and a considerably longer median OS of 18.87 months (95% CI: 15.28–22.45) (40). Consequently, the optimal implementation of 2L ICIs requires precise patient selection to identify those patients who will respond well to ICIs therapy. However, the underlying mechanisms driving this phenomenon need to be elucidated.

In the 2L-ICIs cohort in this study, multivariate Cox regression analysis identified baseline liver metastases as an independent prognostic factor for both PFS and OS. These results align with previous findings that patients with liver metastases are typically associated with a poor prognosis (9, 24, 41, 42). Additionally, multivariate analysis confirmed that 1L-PFS was an independent prognostic factor for both PFS and OS in this cohort. Patients who achieved a 1L PFS of ≥6 months derived greater clinical benefit from subsequent 2L ICIs, a result confirmed by real-world evidence from Zhang et al. (31). This enhanced benefit might be attributed to the fact that patients with a 1L PFS of ≥6 months have a less aggressive tumor phenotype. Additionally, extending 1L-PFS enables critical immune reconstitution, thereby establishing favorable immunological findings for subsequent ICIs therapy. Collectively, these findings identify a 1L-PFS ≥6 months as a clinically actionable biomarker for selecting patients for 2L ICIs therapy. Prospective validation across different cohorts is needed to confirm its utility in guiding stratified treatment strategies, and further research is needed to determine its integration into clinical practice. Therefore, optimizing therapeutic decisions requires the consideration of multiple factors, including disease characteristics, duration of response to 1L therapy, patient preferences, and socioeconomic factors.

The absence of reliable molecular biomarkers for predicting the efficacy of ICIs treatment in ES-SCLC patients is widely recognized. Although PD-L1 expression has emerged as a potential biomarker in other types of cancer (43), its utility in ES-SCLC remains limited. This is largely due to the lower and less consistent expression of PD-L1 in SCLC than in non-small cell lung cancer (NSCLC), making it a less reliable predictor of response to ICIs (44). The variability in PD-L1 expression and the heterogeneity of SCLC tumors further complicate the interpretation of PD-L1 expression in this setting. Emerging biomarkers, such as DLL3 expression, T-cell-inflamed gene expression profiles, and blood tumor mutational burden, show promise in preliminary studies but require validation in prospective ES-SCLC cohorts (45). Predictive biomarker exploration was beyond the scope of this study; however, future prospective research—featuring molecularly characterized cohorts and validated biomarker panels—is needed to optimize immunotherapeutic strategies for patients with ES-SCLC.

The safety analysis revealed comparable tolerability profiles for 2L-ICIs and 2L-non-ICIs when assessing TRAEs excluding irAEs. The 2L ICIs cohort exhibited a favorable safety profile, with only a single case of grade 3 cutaneous toxicity (rash) reported. These findings suggest a reduced susceptibility to severe dose-limiting toxicities in this patient population. This favorable safety profile is clinically significant, given the established links between effective adverse event management and both patient quality of life and treatment adherence. The observed low incidence of severe irAE further indicates that careful patient selection and monitoring can mitigate immunotherapy-related safety concerns. This phenomenon can be explained by two key factors. First, the shorter ICIs treatment duration in the 2L therapy (median of three cycles) may be insufficient to trigger irAEs compared to that in the 1L ICIs group. Second, given the retrospective nature of the study, low-grade irAEs may be under-reported due to inconsistent documentation practices inherent in real-world data.

Although our study provided valuable insights into the efficacy and safety of 2L ICIs therapy in ES-SCLC, it had several limitations. First, the retrospective design and reliance on real-world data may have introduced selection biases and confounding factors, potentially influencing the results. Second, heterogeneous treatment regimens, including various ICIs, chemotherapeutic agents, and combination approaches, introduce significant complexity with potential implications for the interpretation of outcomes. Consequently, future studies should use consistent combined treatment strategies to reduce confounding. Third, we did not perform stratified analyses based on 1L therapy. Further studies are needed to determine the efficacy of cross-line ICIs therapy in subgroups receiving 1L ICIs. Additionally, the lack of effective predictive molecular biomarkers is a significant constraint. Despite these limitations, our findings provided strong evidence for the use of ICIs in ES-SCLC patients after administering 1L therapy. In the future, prospective studies with larger cohorts, standardized treatment protocols, and comprehensive biomarker analyses are needed to validate our findings and identify predictive biomarkers for treatment response. These findings may facilitate precise patient stratification while administering 2L ICIs treatment to patients with ES-SCLC and foster biomarker-driven therapeutic innovations, ultimately improving survival outcomes and quality of life.

## 5 Conclusions

In conclusion, we found that 2L ICIs enhance survival and are safe for use in ES-SCLC patients; therefore, this modality is a viable 2L therapeutic option.

## Data Availability

The original contributions presented in the study are included in the article/supplementary material. Further inquiries can be directed to the corresponding authors.
