# Correction: Real-world outcomes of immune checkpoint inhibitors as second-line therapy for extensive-stage small-cell lung cancer: a multicenter retrospective analysis

**DOI:** 10.3389/fimmu.2025.1708889

**Published:** 2025-10-08

**Authors:** Jiao Zhang, Jia-xing Guo, Ping Li, Xiao-ye Jin, Yan Wang, Lu-jun Zhao

**Affiliations:** ^1^ The Third Department of Medical Oncology, General Hospital of Ningxia Medical University, Yinchuan, Ningxia, China; ^2^ Department of Radiation Oncology, Tianjin Medical University Cancer Institute and Hospital, National Clinical Research Center for Cancer, Tianjin Key Laboratory of Cancer Prevention and Therapy, Tianjin’s Clinical Research Center for Cancer, Tianjin, China; ^3^ Department of Radiation Oncology, The Affiliated Hospital of Inner Mongolia Medical University, Hohhot, Inner Mongolia Autonomous Region, China; ^4^ Department of Pharmacy, General Hospital of Ningxia Medical University, Yinchuan, Ningxia, China; ^5^ College of Clinical Medical, Ningxia Medical University, Yinchuan, Ningxia, China

**Keywords:** extensive-stage small cell lung cancer, immune checkpoint inhibitors, second-line therapy, survival, prognosis

In the **Abstract**, In the **Results** section of the **Abstract**: Line 4: The values “4.14 vs. 2.84” are incorrect. Line 5: The values “4.21 vs. 2.87” are incorrect. This has been corrected to read:

Line 4: The original data has been changed to “4.13 vs. 2.70”. Line 5: The original data has been changed to “4.14 vs. 2.84”.

The original version of this article has been updated.

